# Alzheimer’s Disease Classification Using Population-Referenced Brain Volumetric Percentiles

**DOI:** 10.3390/brainsci16030334

**Published:** 2026-03-20

**Authors:** Jae Hyuk Shim, Hyeon-Man Baek

**Affiliations:** 1 MTech Lab Co., Ltd., Room B1027, 119, Songdo Munhwa-ro, Yeonsu-gu, Incheon 21985, Republic of Korea; 2 Department of Health Sciences and Technology, GAIHST, Gachon University, Incheon 21999, Republic of Korea

**Keywords:** Alzheimer’s disease, MRI, neuroimaging, volumetric analysis, segmentation, structural analysis

## Abstract

**Highlights:**

**What are the main findings?**

- Population-referenced brain volumetric percentiles across 95 regions achieved excellent Alzheimer’s disease classification with AUC values exceeding 0.960 on ADNI internal validation, ADNI test, and independent Korean external validation datasets.
- The minimal validation gap of 0.018 between ADNI and Korean cohorts demonstrates robust model generalization across different populations, scanner protocols, and demographic compositions without requiring dataset-specific retraining.

**What are the implications of the main findings?**

- Percentile-based classification enables individual-level AD diagnosis without requiring longitudinal monitoring or age- and sex-matched control groups, addressing a major barrier to clinical translation of volumetric biomarkers.
- The dual-validation approach with external Korean cohort validation provides strong evidence that automated segmentation combined with population-referenced percentiles can serve as an accessible, cross-population neuroimaging tool for Alzheimer’s disease assessment.

**Abstract:**

**Background/Objectives**: Translating brain volumetric biomarkers to individual-level Alzheimer’s disease (AD) diagnosis remains challenging due to difficulty interpreting raw volumes without longitudinal monitoring or matched controls. We tested a classification model using population-referenced volumetric percentiles to distinguish AD from cognitively normal (CN) subjects and evaluated its generalization across independent cohorts. **Methods**: Brain volumes from 95 regions were extracted using an automated segmentation pipeline and converted to age and sex adjusted percentiles using a reference population (N = 1833). A logistic regression classifier was trained on ADNI subjects (N = 873; AD = 183, CN = 690) split into training (60%), validation (20%), and test (20%) sets. The model was evaluated on two independent validation datasets: the held-out ADNI validation set and an external Korean cohort (N = 72; AD = 36, CN = 36) acquired with different scanner protocols and demographic characteristics. **Results**: The model achieved excellent discrimination across all evaluation sets: ADNI validation (AUC = 0.963, accuracy = 90.3%), ADNI test (AUC = 0.960, accuracy = 89.7%), and Korean external validation (AUC = 0.981, accuracy = 87.5%). The minimal validation gap (0.018) demonstrated robust generalization. Positive coefficients for ventricular regions reflected AD-associated atrophy patterns, while negative coefficients for medial temporal structures indicated their contribution within multivariate patterns distinguishing AD from normal aging. **Conclusions**: Population-referenced brain volumetric percentiles enable accurate AD classification with robust generalization across populations and scanner protocols. By contextualizing individual brain structure relative to normative populations while accounting for age and sex, this approach demonstrates potential for clinical translation as an accessible neuroimaging-based diagnostic tool.

## 1. Introduction

Magnetic resonance imaging (MRI) has established itself as a powerful, non-invasive tool for examining structural brain changes associated with Alzheimer’s disease (AD). Recent advances in automated brain segmentation have made it possible to efficiently extract volumetric data of structures affected by AD from structural MRI scans [1,2,3]. FastSurfer and similar deep learning-based segmentation tools can rapidly segment dozens of brain regions from a single T1-weighted scan, generating comprehensive volumetric profiles with minimal manual intervention [4,5,6]. Through volumetric segmentation, multiple studies have consistently identified significant volumetric differences between AD patients and cognitively normal (CN) individuals, particularly in medial temporal structures such as the hippocampus, entorhinal cortex, and areas exhibiting white matter (WM) hypointensities [7,8,9,10,11,12].

Despite these well-documented differences, clinical translation remains limited due to most findings representing group-level comparisons that lack individualized diagnostic utility [13,14,15]. The primary challenge in clinical implementation of brain volume measurement is that absolute volumetric measurements are difficult to interpret without extensive longitudinal monitoring of structural changes within an individual. Brain volumes can vary substantially across healthy individuals due to factors such as age, sex, genetics, and normal anatomical variation [15,16,17]. As such, measurements of brain volumes such as the hippocampus can provide limited diagnostic information without knowing whether that volume represents normal variation or pathological atrophy for that particular individual [18,19]. Longitudinal monitoring, while valuable, is time-consuming, expensive, and may delay diagnosis until substantial neurodegeneration has already occurred [20,21].

Population-based normative modeling offers a potential solution by contextualizing individual brain measurements against established reference distributions [17,22]. By calculating percentile scores for each brain region, clinicians can identify where an individual’s regional volume falls within the distribution of a healthy population matched for demographic factors, and make conclusions based on the structural deficits related to the pathology [16,17,23]. Additionally, the inclusion of percentiles for less referenced structures can alert the clinician to atrophy in structures related to non-AD-related pathology that could further facilitate diagnosis [24,25].

In this study, we leveraged population-referenced volumetric percentiles across 95 brain regions to develop and validate a classification model for distinguishing AD from CN individuals [26]. We utilized an automated volumetric segmentation pipeline previously validated in a large population study [2,26], processing subjects from the Alzheimer’s disease Neuroimaging Initiative (ADNI) database and an independent Korean cohort [27]. Using the reference population, percentiles were calculated for each of the 95 brain volumes, which were then used to develop and evaluate a classification model for AD classification. The ADNI AD and CN subjects and their percentiles were used to train and test the classification model, then the model performance was evaluated on two independent validation datasets: (1) a held-out ADNI validation set separated from the training set for assessing internal consistency, and (2) an entirely independent Korean cohort acquired with different scanner protocols and representing a distinct demographic population, for external generalizability. Through this approach, we aim to demonstrate the potential of using brain volume percentiles to classify AD in individual subjects.

## 2. Materials and Methods

### 2.1. Subjects

#### 2.1.1. Reference Dataset

Age-stratified normative percentiles were derived from a cognitively normal (CN) reference cohort comprising 1833 individuals aged 21–90 years. All subjects were screened to exclude neurological or psychiatric disorders according to the criteria described in our previous work [26]. High-resolution T1-weighted structural MRI scans were acquired across multiple sites using standardized acquisition protocols. Detailed descriptions of cohort composition, recruitment procedures, and imaging parameters are provided in [26].

#### 2.1.2. ADNI Dataset

T1-weighted structural MRI data of 873 subjects, spanning ages from 50 to 90 years (M = 385, F = 488), were obtained from the ADNI database. The subjects were classified as AD (*n* = 183) and CN (*n* = 690) based on the entry research group and diagnosis parameters associated with each subject. All images were acquired according to standardized ADNI MRI acquisition protocols, which are publicly available and designed to minimize inter-site variability across scanner platforms and field strengths.

Data used in the preparation of this article were obtained from the Alzheimer’s disease Neuroimaging Initiative (ADNI) database (https://adni.loni.usc.edu). The ADNI was launched in 2003 as a public–private partnership, led by Principal Investigator Michael W. Weiner, MD. The original goal of ADNI was to test whether serial magnetic resonance imaging (MRI), positron emission tomography (PET), other biological markers, and clinical and neuropsychological assessment can be combined to measure the progression of mild cognitive impairment (MCI) and early Alzheimer’s disease (AD). The current goals include validating biomarkers for clinical trials, improving the generalizability of ADNI data by increasing diversity in the participant cohort, and providing data concerning the diagnosis and progression of Alzheimer’s disease to the scientific community. For up-to-date information, see https://adni.loni.usc.edu.

#### 2.1.3. Korean Dataset

A total of 72 unique structural T1-weighted MRI images from each subject were obtained from the Gachon University School of Medicine, omitting subjects with a history of alcoholism, depression, and other potential mental illnesses, as well as subjects with visible structural lesions shown in MRI scans. A total of 36 Alzheimer patients were diagnosed with Alzheimer’s through a combination of clinical tests such as the Korean Mini-Mental State Examination (K-MMSE), and through the Clinical Dementia Rating test (score of 0.5 or 1), as well as meeting the Alzheimer’s Criteria proposed by the National Institute of Neurological and Communicative Disorders and Stroke (NINCDS), and the Alzheimer’s Disease and Related Disorders Association (ADRDA). The structural MRI images of the Korean dataset subjects were acquired using the same Siemens MAGNETOM Verio 3T MRI scanner with the following imaging parameters: 1.9 ms TR, 2.9 ms TE, 240 mm FOV, 8° flip angle, 1 × 1 × 1.0 mm voxel size, 176 slices without gaps.

### 2.2. Image Processing

Image processing followed the pipeline described in ref. [26]. The T1-weighted images underwent preprocessing including skull stripping, bias field correction, and spatial normalization. Brain segmentation was performed using FastSurfer [2], an automated deep learning-based tool that segments cortical and subcortical structures. A total of 95 brain regions were extracted from each subject, representing both cortical and subcortical structures. All segmentations underwent visual quality control to ensure accurate processing.

### 2.3. Percentile Score Calculation

For each subject, regional brain volumes were converted to percentile scores using the reference population normative data. Percentiles were calculated using age- and sex-adjusted regression models fitted to the reference population, accounting for normal variation in brain volumes across the lifespan. For each brain region *r*, a linear regression model was fitted to the reference population to estimate the expected volume as a function of age and sex:

$$V_{r,expected}=\beta_{0}+\beta_{1}\cdot Age+\beta_{2}\cdot Sex+\epsilon$$

where $V_{r,expected}$ is the predicted volume for region r, $\beta_{0},\;\beta_{1},\;\beta_{2}$ are regression coefficients, age is in years, sex is binary-encoded (0 = female, 1 = male), and ϵ represents the residual error with standard deviation $\sigma_{r}$.

Each subject’s regional volume was compared to the reference distribution to derive a percentile score (0–100) indicating where the individual’s volume falls relative to the normative population. For each subject i and brain region r, the observed volume $V_{r,i}$ was compared to the normative distribution. The z-score was calculated as:

$$z_{r,i}=\;\frac{V_{r,i}\;-\;V_{r,expected,i}}{\sigma_{r}}$$

where $V_{r,expected,i}$ is the predicted volume for subject i based on their age and sex, and $\sigma_{r}$ is the residual standard deviation from the normative model. The z-score was then converted to a percentile using the cumulative distribution function of the standard normal distribution:

$$P_{r,i}=\Phi \left(z_{r,i}\right)\times 100$$

where Φ is the standard normal cumulative distribution function and $P_{r,i}$ is the percentile score (0–100) for region r in subject i. These percentile scores represent the proportion of the reference population with volumes equal to or less than the observed volume, adjusted for age and sex. Lower percentiles indicate smaller volumes relative to the normative population.

### 2.4. Classification Model Development

All 95 common brain regions available in both ADNI and Korean datasets were included as input features, with each subject represented by a feature vector. $X_{i}=[P_{1,i},P_{2,i},\ldots ,P_{95,i}]$, where $P_{r,i}$ is the percentile score for brain region r. A logistic regression classifier with L2 regularization was employed to estimate the probability of AD classification:

$$P\left(Y=AD|||X\right)=\;\frac{1}{1+\;e^{-(\beta_{0}\;+\;\sum_{r=1}^{95}\beta_{r}X_{r)}}}$$

where Y is the binary class label (AD = 1, CN = 0), $\beta_{0}$ is the intercept term, and $\beta_{r}$ are the learned coefficients for each brain region. Class weights were set inversely proportional to class frequencies ($w_{\mathrm{class}}=N/(2\cdot N_{\mathrm{class}})$) to address the imbalanced AD/CN ratio in the training set. The regularization strength was set to C = 1.0, providing moderate regularization appropriate for the feature-to-sample ratio (95 features, 523 training samples). The maximum number of iterations was set to 1000, and a fixed random seed (42) was used for reproducibility.

The model was trained exclusively on the ADNI training set (N = 523). Prior to model fitting, feature standardization was applied using z-score normalization, where the mean and standard deviation were computed solely from the training set and subsequently applied to all validation and test sets. Five-fold stratified cross-validation was performed within the training set to assess model stability, with each fold maintaining consistent AD/CN proportions. Validation and test sets remained completely unseen during model development, and no hyperparameter tuning, model selection, or coefficient adjustment was performed using validation or test set performance. The model trained on the full training set was applied without modification to all evaluation datasets.

### 2.5. Model Validation and Evaluation

Three independent evaluations were conducted. First, the model was evaluated on the ADNI internal validation set (N = 175), which was held out during training to test model performance on unseen data from the same population and acquisition protocol. Second, the model was evaluated on the Korean cohort (N = 72), representing a completely independent dataset with different scanner protocols and demographic characteristics, with no model retraining or parameter adjustment performed. Third, after validation, the final unbiased performance was assessed on the ADNI test set (N = 175), which remained untouched throughout model development and validation phases.

### 2.6. Statistical Analysis

Model performance was quantified using multiple metrics derived from the confusion matrix. For each evaluation set, predictions were made by applying a decision threshold of 0.5 to the predicted probability. From the confusion matrix containing true positives (TP), true negatives (TN), false positives (FP), and false negatives (FN), we calculated accuracy, sensitivity (TP/[TP + FN]), specificity (TN/[TN + FP]), precision (TP/[TP + FP]), and F1-score.

The area under the receiver operating characteristic curve (AUC-ROC) served as the primary performance metric. The ROC curve was generated by varying the classification threshold across all possible values and plotting the true positive rate against the false positive rate. AUC values range from 0 to 1, with 0.5 indicating chance performance and 1.0 indicating perfect classification. Performance on the training set was assessed using 5-fold stratified cross-validation, with results reported as mean AUC ± standard deviation across folds.

To quantify model generalizability across datasets, the validation gap was computed as the absolute difference between internal ADNI validation and external Korean validation AUC scores (|AUC_ADNI-Val_ − AUC_Korean_|). A validation gap approaching zero indicates the model learned generalizable features rather than dataset-specific patterns, while a larger gap suggests potential overfitting to training population characteristics or acquisition parameters.

The contribution of each brain region to the classification decision was assessed by examining the model coefficients ($\beta_{r}$) obtained from the trained logistic regression model. In logistic regression, each coefficient represents the change in log-odds of AD classification associated with a one-unit increase in the standardized percentile score for that region. After training, coefficients were extracted from the model and ranked by their absolute magnitude. Positive coefficients indicate that lower percentile scores (smaller volumes) in that region increase the probability of AD classification, while negative coefficients indicate that lower percentile scores in that region are associated with CN classification. Since all features were standardized prior to model fitting, coefficient magnitudes are directly comparable across regions, with larger absolute values indicating stronger influence on the classification decision.

## 3. Results

### 3.1. Subject Characteristics

The reference population consisted of 1833 cognitively normal individuals with age ranges of 21–90 years, of which 970 were male, and 863 were female. The ADNI cohort included 690 CN subjects (age: 71.8 ± 7.0 years, 403 females, 287 males, MMSE: 29.1 ± 1.1) and 183 AD subjects (age: 77.3 ± 6.1 years, 85 females, 98 males, MMSE: 22.6 ± 2.6). The Korean cohort included 36 CN subjects (age: 68.5 ± 7.1 years, 17 females, 19 males, K-MMSE: 28.0 ± 1.9) and 36 AD subjects (age: 67.8 ± 7.0 years, 17 females, 19 males, K-MMSE: 18.7 ± 5.9). Detailed demographics are shown in Table 1.

### 3.2. Model Performance

**Figure 1:**
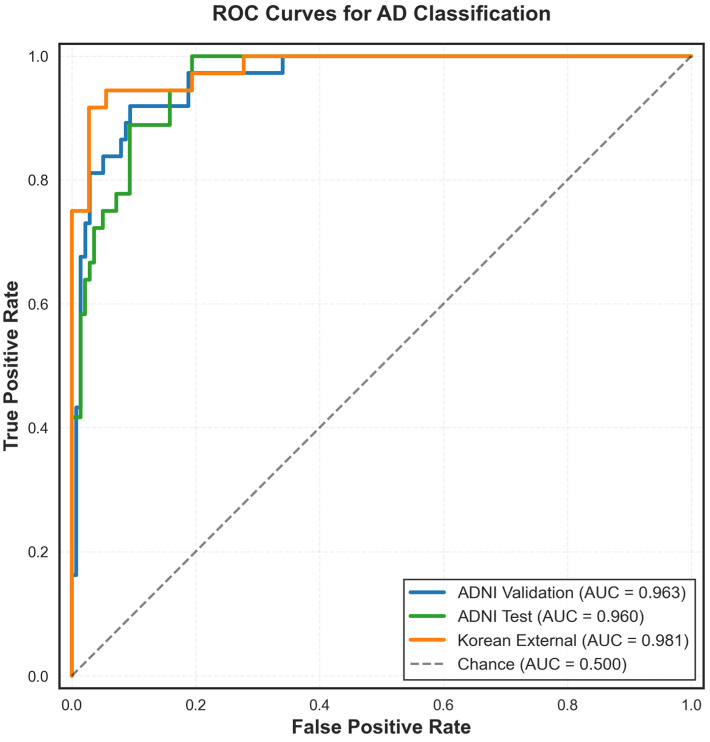
ROC curves for AD classification across evaluation sets. Receiver operating characteristic (ROC) curves showing model performance on three independent evaluation datasets: ADNI validation (blue, AUC = 0.963), ADNI test (green, AUC = 0.960), and Korean external (orange, AUC = 0.981). The diagonal dashed line represents chance performance (AUC = 0.500).

The logistic regression model trained on the ADNI training set (N = 523; AD = 110, CN = 413) achieved a mean AUC of 0.961 ± 0.012 on five-fold stratified cross-validation. Evaluation on the ADNI internal validation set (N = 175; AD = 37, CN = 138) yielded an AUC of 0.963 and an overall accuracy of 90.3%. Sensitivity was 92% (34/37) and specificity was 90% (124/138). Precision was 98% for CN and 71% for AD classification, with F1-scores of 0.94 and 0.80 for CN and AD, respectively. The confusion matrix showed 124 true negatives, 34 true positives, 14 false positives, and 3 false negatives. The ROC curves for AD classification with the ADNI dataset and the Korean dataset are visualized in Figure 1. The performance metrics across the three evaluation datasets (ADNI validation, ADNI test, and Korean external) are visualized in Table 2.

**Figure 2:**
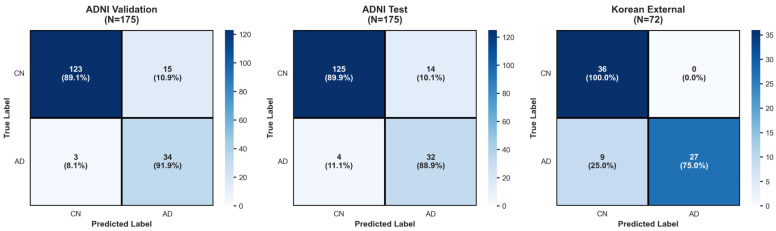
Three-panel confusion matrix heatmaps showing true versus predicted classifications for ADNI validation (N = 175), ADNI test (N = 175), and Korean external (N = 72) datasets. Values indicate the number of subjects with percentages in parentheses.

Evaluation on the held-out ADNI test set (N = 175; AD = 36, CN = 139) yielded an AUC of 0.960 and an accuracy of 89.7%. Sensitivity was 89% (32/36) and specificity was 90% (125/139). Precision was 97% for CN and 70% for AD, with F1-scores of 0.93 and 0.78 for CN and AD, respectively. The confusion matrix showed 125 true negatives, 32 true positives, 14 false positives, and 4 false negatives. On the independent Korean cohort (N = 72; AD = 36, CN = 36), the model achieved an AUC of 0.981 and an overall accuracy of 87.5%. Sensitivity was 75% (27/36) and specificity was 100% (36/36). Precision was 80% for CN and 100% for AD, with F1-scores of 0.89 and 0.86 for CN and AD, respectively. The confusion matrix showed 36 true negatives, 27 true positives, 0 false positives, and 9 false negatives. Confusion matrices of ADNI validation, ADNI test and Korean external are shown in Figure 2.

### 3.3. Cross-Dataset Generalization

The validation gap between internal ADNI validation (AUC = 0.963) and external Korean validation (AUC = 0.981) was 0.018. The mean validation AUC across both independent datasets was 0.972. All three evaluation sets (ADNI validation, Korean external, and ADNI test) yielded AUC values above 0.960.

### 3.4. Feature Importance and Regional Contributions

**Figure 3:**
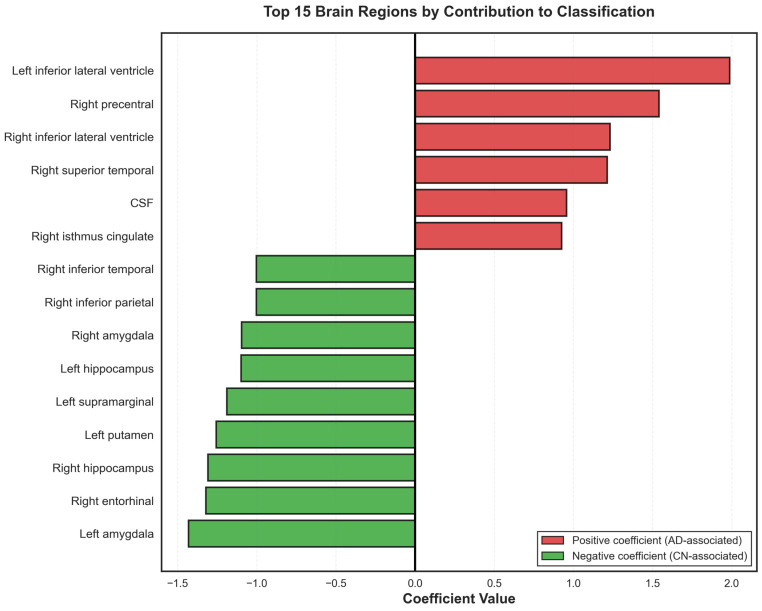
Top 15 contributing brain regions by model coefficient. Horizontal bar chart displaying the 15 brain regions with the largest absolute coefficient values from the logistic regression model. Bars are color-coded: red indicates positive coefficients (AD-associated, higher percentiles increase AD probability) and green indicates negative coefficients (CN-associated, lower percentiles increase CN probability). The left inferior lateral ventricle showed the strongest positive coefficient (β = 1.995), while the left amygdala showed the strongest negative coefficient (β = −1.422).

The brain regions with the largest positive coefficients were the left inferior lateral ventricle (β = 1.995), right precentral gyrus (β = 1.539), right inferior lateral ventricle (β = 1.231), right superior temporal gyrus (β = 1.217), cerebrospinal fluid space (β = 0.956), and the right isthmus cingulate (β = 0.927). The regions with the largest negative coefficients were the left amygdala (β = −1.422), right entorhinal cortex (β = −1.327), right hippocampus (β = −1.292), left putamen (β = −1.252), left supramarginal gyrus (β = −1.192), left hippocampus (β = −1.118), right amygdala (β = −1.105), right inferior parietal (β = −1.002), right inferior temporal (β = −1.002). The coefficients of all brain regions are shown in Table 3, and the 15 brain regions with the highest impact coefficients are shown in Figure 3.

## 4. Discussion

In this study, we developed and validated a classification model for Alzheimer’s disease (AD) versus cognitively normal (CN) subjects using population-referenced brain volumetric percentiles across 95 regions. The model achieved AUC values exceeding 0.960 across three independent evaluation sets: ADNI internal validation (AUC = 0.963), ADNI test (AUC = 0.960), and an independent Korean cohort (AUC = 0.981), with accuracies ranging from 87.5% to 90.3%. The minimal validation gap of 0.018 between ADNI and Korean datasets demonstrates robust generalization across different populations, scanner protocols, and demographic compositions. These results indicate that percentile-based features can capture stable structural patterns of AD across technical and demographic variations, addressing a key limitation in volumetric biomarker translation of interpreting raw brain volumes without group-level comparisons or longitudinal monitoring.

The high classification accuracy across all evaluation sets demonstrates that the combination of percentiles from 95 brain regions can provide sufficient discriminative information to distinguish AD from normal aging. The ADNI datasets showed balanced performance with a sensitivity of 89–92% and specificity of 90%, correctly identifying most AD cases while maintaining low false positive rates. The Korean cohort exhibited different performance characteristics, with perfect specificity (100%, zero false positives) but lower sensitivity (75%, nine false negatives). This pattern in the Korean cohort may be due to several factors. While percentiles account for age-related variation in brain volumes, the smaller age difference between the Korean AD and CN groups (0.7 years) compared to ADNI (5.5 years) may contribute to reduced sensitivity. More importantly, the higher variability in Korean AD K-MMSE scores (18.7 ± 5.9) compared to ADNI (22.6 ± 2.6) suggests greater heterogeneity in disease severity, likely including earlier-stage or milder AD cases with less pronounced structural changes. This disease heterogeneity explains the conservative classification pattern (perfect specificity, lower sensitivity), where the model correctly avoids false positives but misses some milder AD cases that may not yet exhibit sufficient structural changes for reliable percentile-based detection [21,28,29]. Despite these differences, all three evaluation sets yielded AUC values between 0.960 and 0.980, indicating that the percentile-based approach captures fundamental structural patterns of AD that generalize across demographic and technical variations [30,31].

Analysis of model coefficients revealed distinct patterns of regional contributions to classification. The strongest positive coefficients were found for ventricular regions: left inferior lateral ventricle (β = 1.995) and right inferior lateral ventricle (β = 1.231). Ventricular enlargement is a well-established marker of AD progression, resulting from generalized brain atrophy and loss of white matter [32]. As brain parenchyma degenerates, compensatory expansion of ventricular spaces occurs, particularly affecting the temporal horns of the lateral ventricles, which are adjacent to the atrophying hippocampus and medial temporal structures [30,33]. CSF (β = 0.956) also showed a positive coefficient, reflecting global volume loss and cortical atrophy patterns characteristic of AD [34]. The right precentral gyrus (β = 1.539) and right superior temporal gyrus (β = 1.217) also exhibited positive coefficients. The precentral gyrus, housing the primary motor cortex, has been frequently associated with significant reductions in activity and connectivity in AD patients, which could explain the reduced motor and action planning seen in AD patients [35,36]. The superior temporal gyrus plays roles in auditory processing and language comprehension, and its atrophy has been associated with semantic memory deficits and language disturbances observed in AD [37].

In contrast, these traditionally AD-vulnerable medial temporal regions showed negative coefficients: left amygdala (β = −1.422), right entorhinal cortex (β = −1.327), right hippocampus (β = −1.292), and left hippocampus (β = −1.118). The entorhinal cortex serves as the gateway between the hippocampus and neocortex, and is where neurofibrillary tangles first appear in Braak staging [38]. The hippocampus is critical for episodic memory formation and consolidation, and its atrophy directly correlates with memory impairments that constitute the hallmark cognitive symptom of early AD [7,10,30]. The amygdala, involved in emotional processing and memory modulation, also undergoes early atrophy in AD and contributes to behavioral and psychiatric symptoms [39]. Other areas, such as the left putamen (β = −1.252) and inferior parietal regions (right inferior parietal β = −1.002, right inferior temporal β = −1.002) also showed negative coefficients. The putamen is part of the basal ganglia and relatively spared in early AD compared to cortical regions, though it may be affected in later stages or in AD with Lewy bodies [40,41,42]. The inferior parietal cortex, involved in spatial processing and attention, typically shows atrophy in AD and contributes to visuospatial deficits and constructional apraxia commonly observed in patients [43,44]. The negative coefficients for these regions appear counterintuitive but reflect the multivariate nature of the model. When controlling for ventricular enlargement and global atrophy patterns already captured by positive coefficients, isolated atrophy in negative coefficient regions without corresponding ventricular changes may represent normal aging or non-AD pathology [33]. The model may recognize patterns where the combination of ventricular enlargement with specific regional atrophy patterns can distinguish AD from normal aging more effectively than medial temporal volume alone [45].

The study has several limitations. First, the sample size of the Korean external validation cohort (N = 72) was substantially smaller than the ADNI cohort (N = 873), limiting statistical power for detecting subtle differences in generalization performance. Second, while demographic imbalances, particularly in age and sex, were controlled for during percentile generation, comparing cohorts with more matching demographics should further enhance the validity of the results. Third, while two independent validation datasets were employed, both consisted of clinically diagnosed AD and CN subjects, and the model’s performance on subjects with mild cognitive impairment (MCI) or other forms of dementia remains unknown. The cross-sectional design precludes assessment of how early in the disease trajectory the model could detect AD-related changes or whether it could predict progression from CN to AD. Fourth, the Korean validation cohort was the only AD dataset, apart from the ADNI dataset, used to validate the performance of the percentile AD classification model. Fourth, the Korean cohort used different diagnostic criteria and cognitive assessments, which may contribute to differences in model performance between cohorts.

Future work should evaluate the model on larger and more diverse external validation cohorts, including subjects with MCI and non-AD dementias, to assess specificity. However, there is difficulty in the inclusion of MCI due to cases where MCI does not necessarily develop into AD, or the MCI diagnosed in patients is unrelated to AD pathology. As such, longitudinal studies are needed to determine whether the model can identify individuals at risk for AD progression and at what stage of the disease continuum classification becomes reliable.

## 5. Conclusions

In conclusion, population-referenced brain volumetric percentiles across 95 regions enabled accurate classification of AD versus CN subjects, with robust generalization from ADNI to an independent Korean cohort. The minimal validation gap and high performance across datasets acquired with different scanners and protocols demonstrate the potential of this approach for clinical translation. The use of percentiles contextualizes individual brain structure relative to normative populations, providing an interpretable framework that accounts for age and sex without requiring longitudinal monitoring. While further validation in larger and more diverse cohorts is needed, these findings support the feasibility of using population-referenced volumetric percentiles as an accessible neuroimaging-based tool for AD assessment.

## Figures and Tables

**Table 1:** Demographics of cohorts.

Dataset	Group	N	Age (Years)	Male, N (%)	Female, N (%)	MMSE
Reference Population	CN	1833	21–90	970 (52.9)	863 (47.1)	N/A
ADNI Cohort	CN	690	71.8 ± 7.0	287 (41.6)	403 (58.4)	29.1 ± 1.1
	AD	183	77.3 ± 6.1	98 (53.6)	85 (46.4)	22.6 ± 2.6
Korean Cohort	CN	36	68.5 ± 7.1	19 (52.8)	17 (47.2)	28.0 ± 1.9
	AD	36	67.8 ± 7.0	19 (52.8)	17 (47.2)	18.7 ± 5.9

Demographic characteristics for the reference population, ADNI cohort, and Korean cohort, stratified by diagnostic group (CN and AD). Age and cognitive scores are reported as mean ± SD. Gender distribution is reported as a number with a percentage in parentheses. Abbreviations: N, number of subjects; SD, standard deviation; MMSE, Mini-Mental State Examination; AD, Alzheimer’s disease; CN, cognitively normal; N/A, not available.

**Table 2:** Classification performance metrics across evaluation sets.

Evaluation Set	N (AD/CN)	AUC	Accuracy	Sensitivity	Specificity	Precision (AD)	F1-Score (AD)
Training (5-fold CV)	523 (110/413)	0.961 ± 0.012	—	—	—	—	—
ADNI Validation	175 (37/138)	0.963	0.897	0.919	0.891	0.694	0.791
ADNI Test	175 (36/139)	0.960	0.897	0.889	0.899	0.696	0.78
Korean External	72 (36/36)	0.981	0.875	0.75	1	1	0.857

Performance metrics for training (5-fold cross-validation) and three independent evaluation sets. Sample sizes show total subjects with AD/CN counts in parentheses. All evaluation metrics were calculated at a classification threshold of 0.5. Abbreviations: N, number of subjects; AD, Alzheimer’s disease; CN, cognitively normal; AUC, area under the receiver operating characteristic curve; SD, standard deviation; CV, cross-validation.

**Table 3:** Model coefficients for all brain regions.

Region	Coefficient	Region	Coefficient
Left inferior lateral ventricle	1.995	Left choroid plexus	−0.010
Right precentral	1.539	Right pericalcarine	−0.028
Right inferior lateral ventricle	1.231	Right supramarginal	−0.063
Right superior temporal	1.216	Right transverse temporal	−0.081
CSF	0.956	Right rostral middle frontal	−0.089
Left superior temporal	0.929	Right caudate	−0.090
Right isthmus cingulate	0.927	Right cuneus	−0.092
Right cerebral white matter	0.842	Right pars orbitalis	−0.098
Right lateral orbitofrontal	0.739	Left pars orbitalis	−0.113
WM hypointensities	0.717	Left pallidum	−0.158
Left paracentral	0.671	Right cerebellum cortex	−0.162
Right postcentral	0.670	Left ventral DC	−0.166
Left medial orbitofrontal	0.623	Right posterior cingulate	−0.179
Right pallidum	0.620	Left-Thalamus	−0.209
Left cerebellum cortex	0.488	Left precentral	−0.241
Right-Thalamus	0.422	Right insula	−0.264
Left pars opercularis	0.391	Left lateral orbitofrontal	−0.283
Left superior parietal	0.383	3rd Ventricle	−0.299
Left accumbens area	0.381	Left precuneus	−0.302
Left caudal anterior cingulate	0.365	Left parahippocampal	−0.304
Right pars triangularis	0.364	Right medial orbitofrontal	−0.319
Right rostral anterior cingulate	0.347	Left transverse temporal	−0.320
Left inferior parietal	0.345	Left postcentral	−0.471
Left insula	0.336	Left rostral anterior cingulate	−0.484
Left cuneus	0.329	Right choroid plexus	−0.520
Brain Stem	0.308	Right superior parietal	−0.556
Right lateral occipital	0.299	Left cerebellum white matter	−0.584
Right caudal middle frontal	0.273	Left posterior cingulate	−0.611
Right fusiform	0.272	Left entorhinal	−0.619
Right accumbens area	0.261	Right lateral ventricle	−0.655
Left pars triangularis	0.247	Right precuneus	−0.667
Right lingual	0.228	Left middle temporal	−0.671
Right superior frontal	0.225	Left inferior temporal	−0.679
Right pars opercularis	0.213	Left fusiform	−0.694
Left cerebral white matter	0.199	Left lingual	−0.733
Right cerebellum white matter	0.196	Left isthmus cingulate	−0.858
Right putamen	0.161	Left caudal middle frontal	−0.886
4th Ventricle	0.149	Right inferior parietal	−1.002
Right parahippocampal	0.147	Right inferior temporal	−1.002
Left superior frontal	0.135	Right amygdala	−1.105
Left rostral middle frontal	0.132	Left hippocampus	−1.119
Right middle temporal	0.111	Left supramarginal	−1.192
Right paracentral	0.098	Left putamen	−1.252
Left pericalcarine	0.090	Right hippocampus	−1.292
Left caudate	0.083	Right entorhinal	−1.327
Right ventral DC	0.082	Left amygdala	−1.422
Right caudal anterior cingulate	0.050		
Left lateral ventricle	0.017		
Left lateral occipital	0.004		

All 95 brain regions ranked by absolute coefficient magnitude. Positive coefficients indicate that higher percentile scores increase AD classification probability; negative coefficients indicate that lower percentile scores increase AD classification probability. All features were standardized, making coefficients directly comparable. Abbreviations: β, model coefficient; CSF, cerebrospinal fluid; ventral DC, ventral diencephalon; WM, white matter.

## Data Availability

The data presented in this study are available on request from the corresponding author due to institutional privacy and information security considerations.

## Abbreviations

The following abbreviations are used in this manuscript:

AD	Alzheimer’s disease
ADNI	Alzheimer’s Disease Neuroimaging Initiative
ADRDA	Alzheimer’s Disease and Related Disorders Association
AUC	Area Under Curve
CN	Clinically Normal
CSF	Cerebrospinal Fluid
DC	Diencephalon
FN	False Negatives
FP	False Positives
MCI	Mild Cognitive Impairment
MMSE	Mini-Mental State Examination
MRI	Magnetic Resonance Imaging
NINCDS	National Institute of Neurological and Communicative Disorders and Stroke
ROC	Receiver Operating Characteristic
TN	True Negatives
TP	True Positives
WM	White Matter
